# Supplementary material for: A system for inducible mitochondria-specific protein degradation in vivo
Source: Nat Commun. 2024 Feb 16;15:1454. doi: 10.1038/s41467-024-45819-6 (PMC10873288; doi:10.1038/s41467-024-45819-6)
Supplement: Supplementary file 1 — Supplementary Information [file 41467_2024_45819_MOESM1_ESM.pdf]

Supplementary Information

A system for inducible mitochondria-specific protein degradation *in vivo*

Swastika Sanyal[1]*, Anna Kouznetsova[2], Lena Ström[2], Camilla Björkegren[2]*

Affiliations:

[1]Karolinska Institutet, Department of Biosciences and Nutrition, Neo, Hälsovägen 7c, 141 83 Huddinge, Sweden.

[2]Karolinska Institutet, Department of Cell and Molecular Biology, Biomedicum, Tomtebodavägen 16, 171 77 Stockholm, Sweden.

*Corresponding authors. Email: swastika.sanyal@ki.se, camilla.bjorkegren@ki.se

Supplementary Figure 1

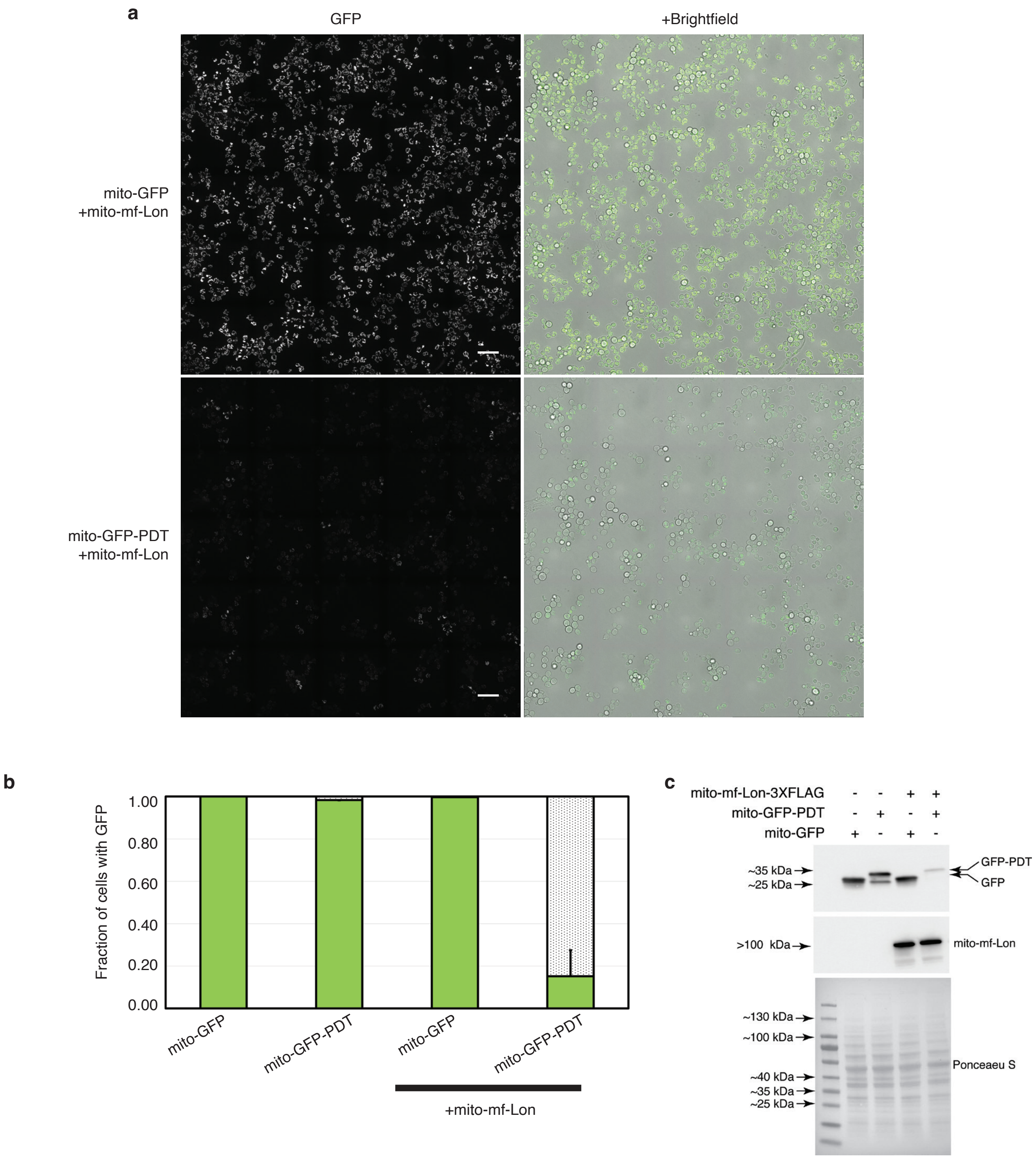

Supplementary Figure 1. **mf-Lon induces mitochondrial GFP-PDT degradation. a,** Large field image of the GFP signal in mito-GFP mito-mf-Lon, and mito-GFP-PDT mito-mf-Lon cells. Scale bar = 20 µm. Cells were grown in minimal dextrose medium and imaged when they reached deceleration growth phase. **b,** Fraction of cells with mitochondrial GFP in the indicated strains. Error bars represent standard deviation from 3 independent clones. Cells were grown in minimal medium and examined 16-18h after inoculation (overnight culture). **c,** Western blot of whole cell extracts from strains shown in **b**, representative of two independent experiments. .

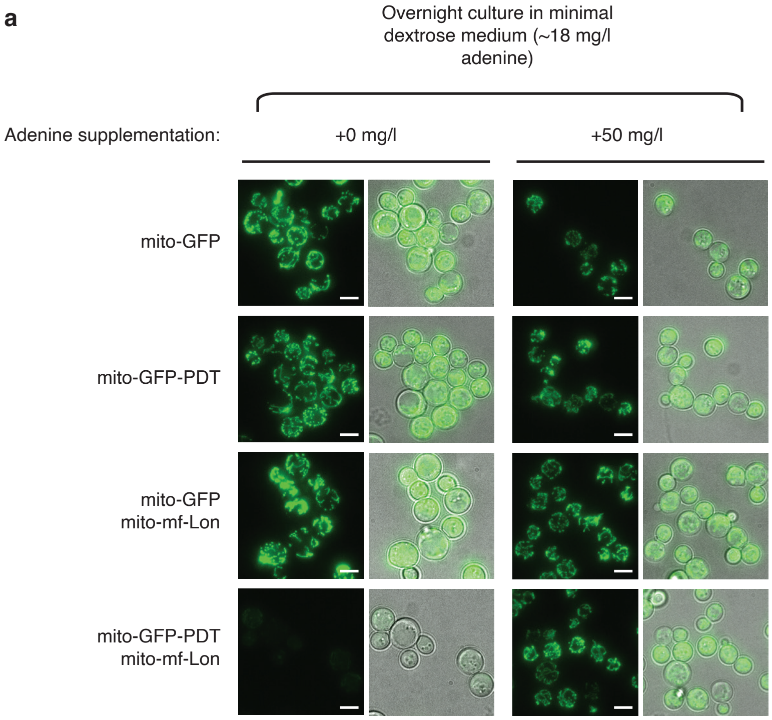

Supplementary Figure 2. Adenine addition prevents mitochondrial GFP-PDT degradation by mf-Lon in minimal medium. a, Indicated strains growing in standard minimal dextrose medium (comprising ~18 mg/l adenine) were left untreated (left panel), or supplemented with adenine (right panel). Representative images of an overnight culture are shown. Scale bar = 5 µm. Images are representative of three independent experiments.

Supplementary Figure 3

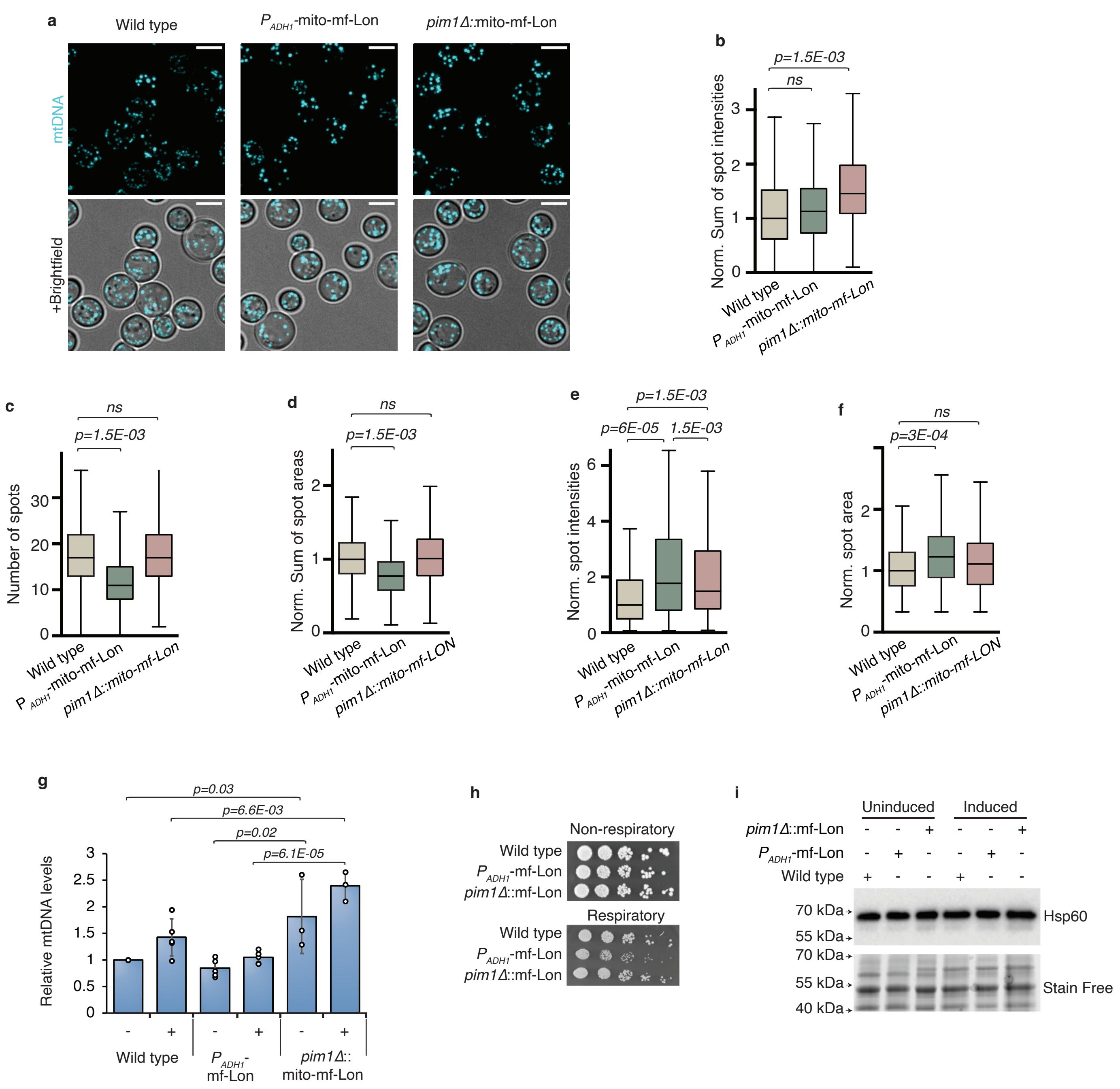

Supplementary Figure 3. Visualization of mitochondrial nucleoids in wild type, *P_{ADH1}*- mito-mf-Lon, and *pim1*Δ::mito-mf-Lon cells. a, Representative images of Sybr Green I-stained mtDNA in indicated strains grown overnight in standard minimal medium. **(b-f)** Quantification of mtDNA spots detected by Sybr Green I. Sum of spot intensities in the cell, representing total mitochondrial DNA content per cell (**b**), number of spots, representing number of nucleoids per cell (**c**), sum of spot areas (**d**), spot intensities (**e**), and area of individual spots (**f**)**,** were compared by hierarchical resampling from three independent experiments. At least 630 cells from each sample were analyzed. Boxes show interquartile range and whiskers at 10% and 90% range. Values in **b**, **d**, **e**, and **f**, were normalized to the wild type median. P-values (p)<0.05 were considered significant (ns = not significant). **g,** WT, *P_{ADH1}*-mito-mf-Lon, and *pim1*Δ::mito-mf-Lon

cells were induced for PDT degradation, and the levels of mtDNA relative to uninduced sample of wild type cells were determined by quantitative real time PCR. The data represent averages with error bars indicating standard deviation from at least three independent experiments. An unpaired, two-tailed students t-test was performed to determine the confidence interval. **h,** Growth test of indicated strains. Five-fold serial dilutions of exponentially growing cells were spotted on agar plates with non-respiratory (glucose) and respiratory (glycerol) media, incubated at 30 degrees for 3 days before imaging. **i.** Whole cell extracts from indicated cells grown under inducing conditions were analyzed by Western blotting for the mitochondrial stress marker Hsp60.

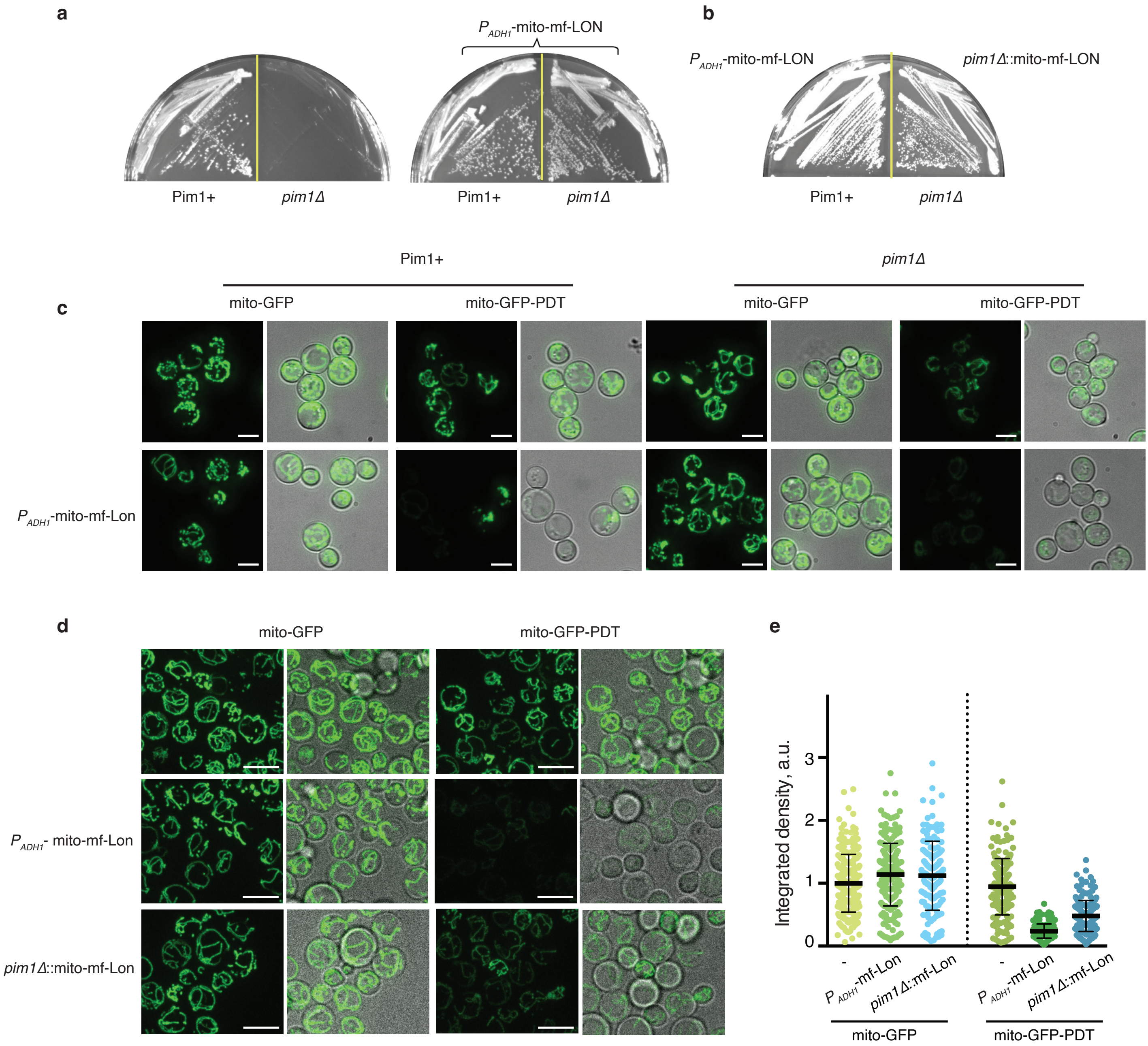

Supplementary Figure 4. Lon-induced PDT degradation occurs independently of yeast endogenous Lon, Pim1. (a-b), mf-Lon rescues respiratory defect of *pim1*Δ cells. **a,** Strains with or without wild type *PIM1*, either independently or co-expressing P_{ADH1}-mito-mf- Lon, were grown on solid respiratory medium, and imaged after incubation at 30˚ C for 4 days. **b,** Same as **a**, but with strains that expressed mf-Lon from the *ADH1* promoter, or from the *PIM1* locus are indicated. **(c-d)** PDT-degradation is specific to mf-Lon. **c,** Cells expressing mito- GFP or mito-GFP-PDT, either independently or co-expressing P_{ADH1}-mito-mf-Lon, in the presence or absence of wild type *PIM1* were grown overnight in standard minimal medium and examined for GFP-PDT degradation. **d,** Cells expressing mito-GFP or mito-GFP-PDT, either independently or

co-expressing P_{ADH1}-mito-mf-Lon, or *pim1*Δ::mito-mf-Lon were examined for PDT-degradation as in **c**. Scale bar=5µm. **e,** Quantification of GFP signal in the cells shown in **d**, expressed as arbitrary units (a.u.). Each dot represents an individual cell. Data depict median and interquartile range and are normalized to the median intensity in mito-GFP strain. At least 150 cells from each sample were examined over two independent experiments.

Supplementary Figure 5

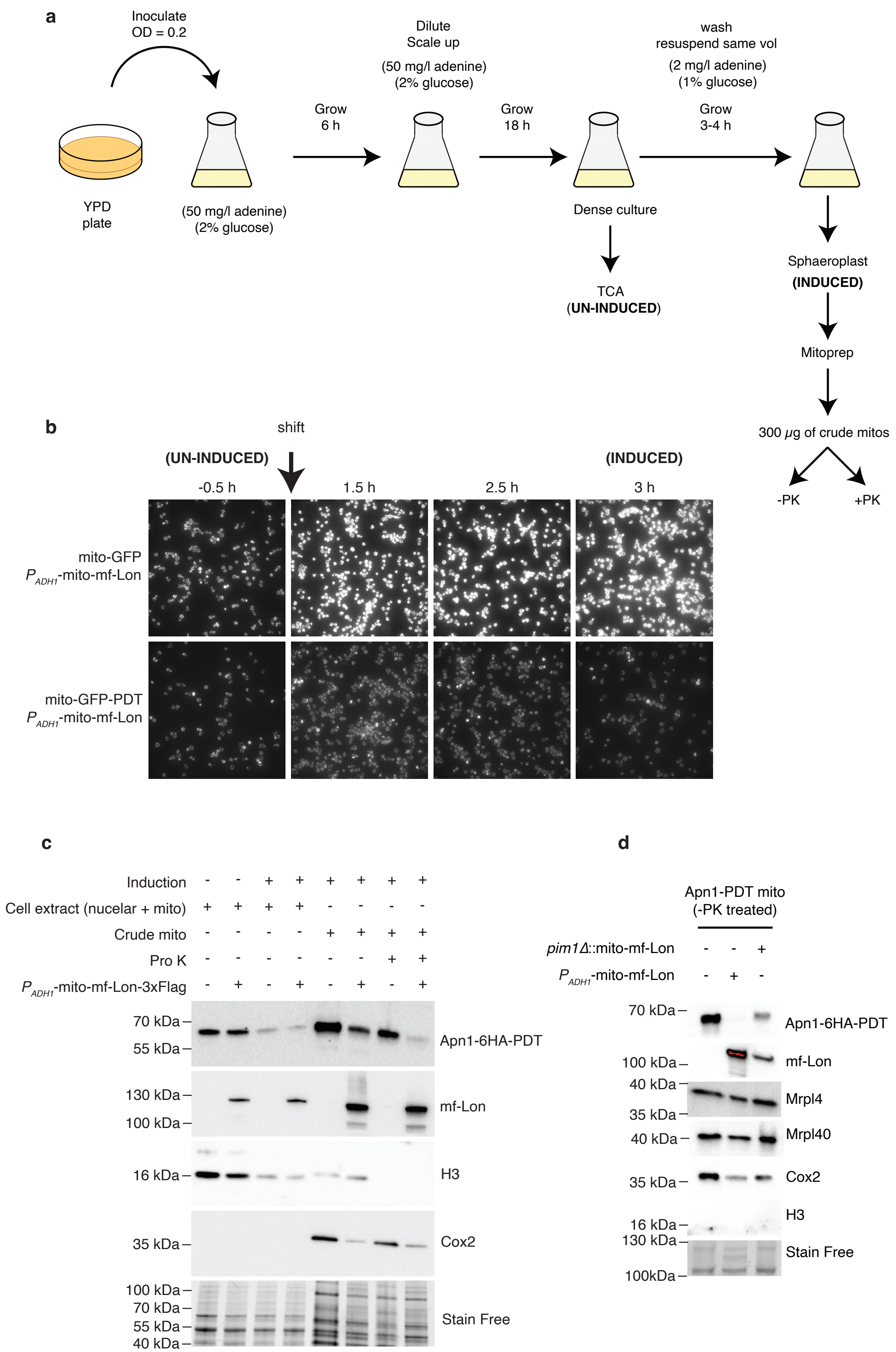

Supplementary Figure 5. mf-Lon protease degrades yeast endogenous mitochondrial proteins tagged with PDT. a, Schematic of induction experiment (also see Materials and Methods for details). **b,** Representative images of P_{ADH1}-mito-mf-Lon cells expressing mito-GFP or mito-GFP-PDT cells imaged 30 mins before (un-induced) and up to 3 hours after the shift in inducing medium (induced), grown in parallel with the experimental strains, to ensure that the media induced PDT-degradation. **c,** Cells expressing Apn1-PDT with or without P_{ADH1}-mito-mf-Lon were induced for PDT-degradation as shown in **a**. Cell extracts from uninduced and induced cultures, and mitochondria from induced samples, were analyzed by Western blotting for indicated proteins. Proteinase K treatment was performed on mitochondria to remove extra-mitochondrial contamination. **d,** Mitochondrial samples as shown in Figure 4a were analysed by Western blotting for additional mitochondrial and nuclear proteins. Absence of H3 indicates a cleaner mitochondrial preparation than in **c**. Stain free image serves as a loading control. The sizes of the proteins are: Apn1-PDT (anti-HA), ~60 kDa; mf-Lon (anti-Flag), ~100 kDa; H3 (anti-H3), ~15 kDa; Cox2 (anti-MTCO2), ~35 kDa, Mrpl4 (anti-Mrpl4), ~44 kDa; Mdh1 (anti-Mdh1), 35.6 kDa; and Mrpl40 (anti-Mrpl40), ~40 kDa.

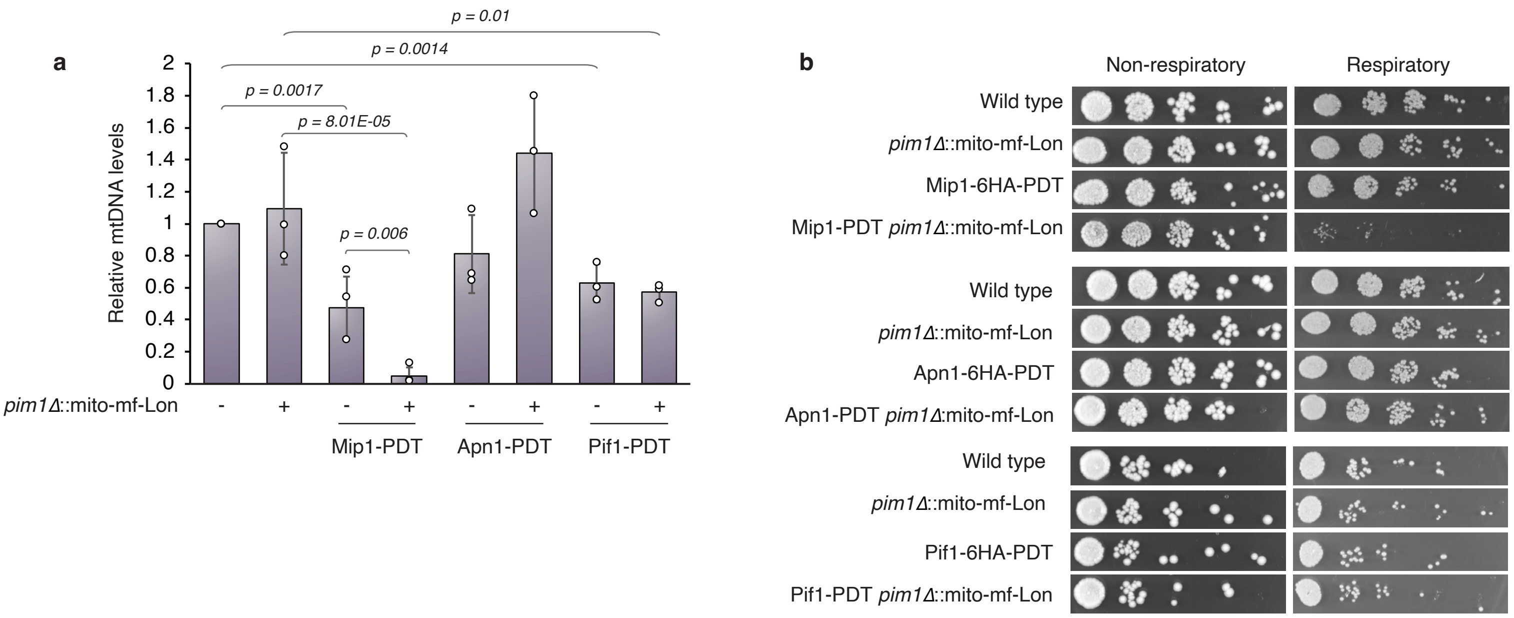

Supplementary Figure 6. Functional consequences of mf-Lon-dependent PDT degradation of endogenous yeast proteins. a, Mitochondrial DNA copy number in cells expressing *pim1*Δ::mito-mf-Lon combined with Apn1-PDT, Pif1-PDT or Mip1-PDT, grown in minimal medium for ~20 h. Relative levels of mtDNA (measured by the mitochondrial *COX2* and nuclear *ACT1* genes) as determined by quantitative real time PCR, and normalized to the wild type samples. The data represent averages with error bars indicating standard deviation from three independent experiments. Unpaired, two-tailed students t-test was performed to determine the confidence interval. P-values < 0.05 were considered significant and are indicated. **b,** Growth test of strains shown in **a**. Five-fold serial dilutions of exponentially growing cells were spotted on agar plates with non-respiratory (glucose) and respiratory (glycerol) media and imaged after 3-days of incubation at 30 degrees.

Supplementary Table 1.

All strains listed were derived from *RAD5* W303 strain, CB67 (*MATa, ade2-1, trp1-1, can1-100, leu2-3,112, his3-11,15, ura3, GAL, psi+, RAD5*), denoted wild type in the manuscript.

**Name**	**Identifier**	**Genotype**
mito-GFP	CB3699	*trp1-1::TRP1-P_{ADH1}-MTS ($SU9^{1-69}$)-GFP-T_{ADH1} (Yiplac204)*
mito-GFP-PDT	CB3701	*trp1-1::TRP1-P_{ADH1}-MTS ($SU9^{1-69}$)-GFP-PDT#3-T_{ADH1} (Yiplac204)*
mito-GFP<br>mito-mf-Lon	CB3713	*trp1-1::TRP1-P_{ADH1}-MTS ($SU9^{1-69}$)-GFP-T_{ADH1} (YIplac204), leu2-3::LEU2-P_{ADH1}-MTS ($SU9^{1-69}$)-MF-LON-6xHIS-3xFLAG-T_{ADH1} (YIplac128)*
mito-GFP-PDT<br>mito-mf-Lon	CB3714	*trp1-1::TRP1-P_{ADH1}- MTS ($SU9^{1-69}$)-GFP-PDT#3-T_{ADH1} (YIplac204), leu2-3::LEU2-P_{ADH1}- MTS ($SU9^{1-69}$)- MF-LON -6xHIS-3xFLAG -T_{ADH1} (YIplac128)*
cyto-GFP	CB3767	*trp1-1::TRP1-P_{ADH1}-GFP-T_{ADH1} (Yiplac204)*
cyto-GFP-PDT	CB3769	*trp1-1::TRP1-P_{ADH1}-GFP-PDT#3-T_{ADH1} (Yiplac204)*
cyto-GFP<br>mito-mf-Lon	CB3782	*trp1-1::TRP1-P_{ADH1}-GFP-T_{ADH1} (Yiplac204), leu2-3::LEU2-P_{ADH1}- MTS ($SU9^{1-69}$)- MF-LON -6xHIS-3xFLAG -T_{ADH1} (YIplac128)*
cyto-GFP-PDT<br>mito-mf-Lon	CB3783	*trp1-1::TRP1-P_{ADH1}-GFP-PDT#3-T_{ADH1} (Yiplac204), leu2-3::LEU2-P_{ADH1}- MTS ($SU9^{1-69}$)-MF-LON -6xHIS-3xFLAG -T_{ADH1} (YIplac128)*
mito-GFP<br>mito-mf-Lon-mCherry	CB3726	*trp1-1::TRP1-P_{ADH1}- MTS ($SU9^{1-69}$)-GFP-PDT#3-T_{ADH1} (YIplac204), leu2-3::LEU2-P_{ADH1}- MTS ($SU9^{1-69}$)- MF-LON -mCherry-T_{ADH1}-kanMx4 (pFA6a-link-yomCherry-Kan)*
mito-GFP-PDT<br>mito-mf-Lon-mCherry	CB3727	*trp1-1::TRP1-T_{ADH1}- MTS ($SU9^{1-69}$)-GFP-P_{ADH1} (YIplac204), leu2-3::LEU2-P_{ADH1}-MTS($SU9^{1-69}$)-*

		MF-LON -mCherry-T_{ADH1}-kanMx4 (pFA6a-link-yomCherry-Kan)
*pim1*Δ<br>mito-GFP	CB3786	*pim1Δ::natMX4 (pCloneNat1), trp1::TRP1-P_{ADH1}-MTS ($SU9^{1-69}$)-GFP-T_{ADH1} (Yiplac204)*
*pim1*Δ<br>mito-GFP-PDT	CB3787	*pim1Δ::natMX4 (pCloneNat1),, trp1-1::TRP1-P_{ADH1}-MTS ($SU9^{1-69}$)-GFP-PDT#3-T_{ADH1} (Yiplac204)*
*pim1*Δ<br>mito-GFP<br>mito-mf-Lon	CB3790	*pim1Δ::natMX4 (pCloneNat1), trp1-1::TRP1-P_{ADH1}-MTS ($SU9^{1-69}$)-GFP-T_{ADH1} (YIplac204), leu2-3::LEU2-P_{ADH1}-MTS ($SU9^{1-69}$)- MF-LON -6xHIS-3xFLAG-T_{ADH1} (YIplac128)*
*pim1*Δ<br>mito-GFP-PDT<br>mito-mf-Lon	CB3791	*pim1Δ::natMX4 (pCloneNat1), trp1-1::TRP1-P_{ADH1}-MTS ($SU9^{1-69}$)-GFP-PDT#3-T_{ADH1} (YIplac204), leu2-3::LEU2-P_{ADH1}-MTS ($SU9^{1-69}$)-MF-LON -6xHIS-3xFLAG-T_{ADH1} (YIplac128)*
*pim1*Δ::mito-mf-Lon<br>mito-GFP	CB3921	*trp1-1::TRP1-P_{ADH1}-MTS($SU9^{1-69}$)-GFP-T_{ADH1} (Yiplac204), pim1Δ::MTS($SU9^{1-69}$)- MF-LON -6xHIS-3xFLAG-LEU2*
*pim1*Δ::mito-mf-Lon<br>mito-GFP-PDT	CB3922	*trp1-1::TRP1-P_{ADH1}- MTS ($SU9^{1-69}$)-GFP-PDT#3-T_{ADH1} (Yiplac204), pim1Δ::MTS ($SU9^{1-69}$)- MF-LON -6xHIS-3xFLAG -LEU2*
pim1Δ::mito-mf-Lon	CB3923	*pim1Δ::MTS ($SU9^{1-69}$)- MF-LON -6xHIS-3xFLAG-LEU2*
Apn1-PDT	CB3876	*APN1-6xHA-PDT#3-kanMX4*
Apn1-PDT<br>P_{ADH1}-mito-mf-Lon	CB3877	*APN1-6xHA-PDT#3-kanMX4, leu2-3::LEU2-P_{ADH1}-MTS ($SU9^{1-69}$)- MF-LON -6xHIS-3xFLAG-T_{ADH1} (YIplac128),*
Apn1-PDT<br>*pim1*Δ::mito-mf-Lon	CB3954	*APN1-6HA-PDT#3-kanMX4, pim1Δ:: MTS ($SU9^{1-69}$)- MF-LON N-6xHIS-3xFLAG-LEU2*
Pif1-PDT	CB3871	*PIF1-6xHA-PDT#3-kanMX4*
Pif1-PDT<br>*pim1*Δ::mito-mf-Lon	CB3955	*PIF1-6HA-PDT#3-kanMX4, pim1Δ::MTS ($SU9^{1-69}$)- MF-LON -6xHIS-3xFLAG-LEU2*
Mip1-PDT	CB4086	*MIP1-6xHA-PDT#3-kanMX4*

Mip1-PDT *pim1*Δ::mito-mf-Lon	CB4090	*MIP1-6HA-PDT#3-kanMX4, pim1Δ:: MTS (SU9$^{1-69}$)- MF-LON N-6xHIS-3xFLAG-LEU2*
P_{ADH1}-mito-mf-Lon	CB3730	*leu2-3::LEU2-P_{ADH1}-MTS (SU9$^{1-69}$)- MF-LON - 6xHIS-3xFLAG -T_{ADH1} (YIplac128)*

Supplementary Table 2.
Primers used in the study.

a. PDT tagging primers:

Name	5'-3' sequence
APN1_6HA_HIS3_up	ATAATGATATCTTGTCACAAATGACAAAGAAGAGGAAC ACTAAGAAAGAA TCCGGTTCTGCTGCTAGATA
APN1_6HA_HIS3_dw	AGATAATCTACAAAAATTGATTACGTATTTAAAATTCT TCTCGCTTCTCA CTTTACAACACTCCCTTCGTGC
PIF1_6HA_HIS3_up	TGGTTTCCGACGAACCTCGTGGTCAGGATACCGAAGA CCACATCTTAGAA TCCGGTTCTGCTGCTAGATA
PIF1_6HA_HIS3_dw	GATTATTATAGCAGTTTGTATTCTATATAACTATGTGT ATTAATATGTAC CTTTACAACACTCCCTTCGTGC
MIP1_6HA_HIS3-up	ATAGAAATTTGGTTGAGCTGGAAAGGGACATTACTAT TTCTAGAGAGTAC TCCGGTTCTGCTGCTAGATA
MIP1_6HA_HIS3-dw	TAATGTGCTGTATATATAAATACAAATGCGAAAGCTA ATGCAGATTTTGC CTTTACAACACTCCCTTCGTGC
*6HA_PDT_up	CGGACTATGCAGGATCCTATCCATATGACGTTCCAGA TTACGCTTCTAGCGCTGCTAACAAGAACGAAG
*6HA_PDT_dw	CTTTACAACACTCCCTTCGTGCTTGGGACTTCAGAACT TCCAGTAAGACTGCGAAGAAGTCCAAAGCTGG

*The PDT-kanMX4 cassette was amplified using CD436 as a template.

b. Primers for deleting *PIM1*, used with pClonNat1 plasmid

Name	5'-3' sequence
Pim1_SP6 Fw	TTTTCTTTTGGTTTTCGAGGTGCCTGAACGAAAAGATTTGCAAA TAAAGC CACATACGATTTAGGTGACAC
Pim1_T7 RV	AAATATTTACAGAATGTTTAAACAGGTATTTAATCCATTTAGA TGAAAAGAATACGACTCACTATAGGGAG

c. Primers for creating *pim1*Δ::mito-mf-Lon strains, used with YIplac128-mt-mf-Lon (CD429) plasmid

Name	5'-3' sequence
pim1::mf-Lon up	TTTTCTTTTGGTTTTCGAGGTGCCTGAACGAAAAGATTTGCAAATAAAGCATGGCTTCTACTAGAGTTTTGG
pim1::mf-Lon dw	AAATATTTACAGAATGTTTAAACAGGTATTTAATCCATTTAGATGAAAAGGGCCAACTGTGGGAATACTC

d. qPCR primers

Name	5'-3' sequence
Cox2 qPCR fw	GTATTAAAGTTGATGCTACTCCTGG
Cox2 qPCR rv	GCTTCGATCTTAATTGGCATATTTG
Act1 qPCR fw	CAGGTATTGCCGAAAGAATG
Act1 qPCR rv	TAGTCAAAGAAGCCAAGATAGA

Supplementary Notes

Choice of the PDT tag

Most bacterial species employ more than one protease to degrade the ssrA tag, and owing to the evolutionary conservation, proteases display inter-species promiscuity in tag detection, making controllable protein degradation challenging [1–5].

Since the *Escherichia coli* (*E. coli*) Lon can degrade mf-Lon ssrA tag, and can functionally complement the yeast Lon, we aimed to use a variant of mf-Lon ssrA tag that is unidentifiable by the *E. Coli* -Lon [pdt#3 (AANKNEENTNEVPTFMLNAGQANRRRV), developed by Cameron and Collins [4]], to avoid interference by yeast endogenous Lon [3,4,6]. Throughout the manuscript, we referred to the pdt#3 tag as PDT.

Optimization of PDT degradation

We considered three parameters to mimic diauxic shift and PDT degradation; high cell concentration, glucose concentration and adenine concentration.

In synthetic minimal medium which contains 18.94 mg/l adenine, a W303 strain which is an adenine auxotroph starts decelerating cell growth upon reaching an OD_{600} ~4-5. Therefore, cells were usually resuspended in the inducing medium at a cell concentration of OD higher than 4.

Cells were grown in 1% glucose to shift cellular metabolism towards respiration, which occurs during diauxic shift [7].

Finally, we chose 2 mg/l of adenine for PDT degradation since this concentration allowed cell division, as judged by budding index, in contrast to complete deprivation of adenine (0 mg/l) which, although inducing PDT degradation faster, impaired cellular growth.

References:

1. Gottesman, S., Roche, E., Zhou, Y. & Sauer, R. T. The ClpXP and ClpAP proteases degrade proteins with carboxy-terminal peptide tails added by the SsrA-tagging system. *Gene Dev* 12, 1338–1347 (1998).

2. Lies, M. & Maurizi, M. R. Turnover of Endogenous SsrA-tagged Proteins Mediated by ATP-dependent Proteases in Escherichia coli *. *J Biol Chem* 283, 22918–22929 (2008).

3. Gur, E. & Sauer, R. T. Evolution of the ssrA degradation tag in Mycoplasma: specificity switch to a different protease. *Proc Natl Acad Sci U S A* 105, 16113–16118 (2008).

4. Cameron, D. E. & Collins, J. J. Tunable protein degradation in bacteria. *Nature biotechnology* 32, 1276–1281 (2014).

5. Butzin, N. C. & Mather, W. H. Crosstalk between Diverse Synthetic Protein Degradation Tags in Escherichia coli. *Acs Synth Biol* 7, 54–62 (2018).

6. Teichmann, U. *et al.* Substitution of PIM1 protease in mitochondria by Escherichia coli Lon protease. *J Biol Chem* 271, 10137–10142 (1996).

7. Bartolomeo, F. D. *et al.* Absolute yeast mitochondrial proteome quantification reveals trade-off between biosynthesis and energy generation during diauxic shift. *Proc Natl Acad Sci U S A* 117, 7524–7535 (2020).
