# Supplementary material for: A system for inducible mitochondria-specific protein degradation in vivo
Source: Nat Commun. 2024 Feb 16;15:1454. doi: 10.1038/s41467-024-45819-6 (PMC10873288; doi:10.1038/s41467-024-45819-6)
Supplement: Supplementary file 3 — Reporting Summary [file 41467_2024_45819_MOESM3_ESM.pdf]

Corresponding author(s): Swastika Sanyal, Camilla Björkegren

Last updated by author(s): Jan 23, 2024

Reporting Summary

Nature Portfolio wishes to improve the reproducibility of the work that we publish. This form provides structure for consistency and transparency in reporting. For further information on Nature Portfolio policies, see our Editorial Policies and the Editorial Policy Checklist.

Statistics

For all statistical analyses, confirm that the following items are present in the figure legend, table legend, main text, or Methods section.

n/a	Confirmed	
☐	☒	The exact sample size (*n*) for each experimental group/condition, given as a discrete number and unit of measurement
☐	☒	A statement on whether measurements were taken from distinct samples or whether the same sample was measured repeatedly
☐	☒	The statistical test(s) used AND whether they are one- or two-sided<br>*Only common tests should be described solely by name; describe more complex techniques in the Methods section.*
☐	☒	A description of all covariates tested
☒	☐	A description of any assumptions or corrections, such as tests of normality and adjustment for multiple comparisons
☐	☒	A full description of the statistical parameters including central tendency (e.g. means) or other basic estimates (e.g. regression coefficient) AND variation (e.g. standard deviation) or associated estimates of uncertainty (e.g. confidence intervals)
☐	☒	For null hypothesis testing, the test statistic (e.g. *F*, *t*, *r*) with confidence intervals, effect sizes, degrees of freedom and *P* value noted<br>*Give P values as exact values whenever suitable.*
☒	☐	For Bayesian analysis, information on the choice of priors and Markov chain Monte Carlo settings
☒	☐	For hierarchical and complex designs, identification of the appropriate level for tests and full reporting of outcomes
☒	☐	Estimates of effect sizes (e.g. Cohen's *d*, Pearson's *r*), indicating how they were calculated

Our web collection on statistics for biologists contains articles on many of the points above.

Software and code

Policy information about availability of computer code

Data collection	NIS-Elements Imaging Software (Nikon Instruments, Tokyo, Japan)<br>LAS X Imaging Software 4.5.0 (Leica microsystems)<br>Zen Imaging software v.3.1 (Zeiss)<br>Biorad Chemi-doc<br>Bio-Rad CFX Real-Time PCR Software v 2.3
Data analysis	1. Microscopy images<br>NIS-Elements Viewer<br>Fiji (Version 2.9.0/1.52p)<br>FeatureJ plugin (http://imagescience.org/meijering/software/featurej/)<br>YeastSpotter (http://yeastspotter.csb.utoronto.ca)<br><br>2. Western Blot images<br>ImageLab Version 6<br><br>3. Real Time PCR<br>CFX Maestro Software (Bio-Rad) Version 2.3<br><br>4. Sequence editor<br>SnapGene Viewer Version 6.2

5. Statistical analysis and graphing
RStudio (http://www.rstudio.com)
GraphPad Prism 9.0 (www.graphpad.com)
Microsoft Excel Version 16.79.1(23111614)

6. Data Compilation and presentation
Adobe Illustrator CS 2024

For manuscripts utilizing custom algorithms or software that are central to the research but not yet described in published literature, software must be made available to editors and reviewers. We strongly encourage code deposition in a community repository (e.g. GitHub). See the Nature Portfolio guidelines for submitting code & software for further information.

Data

Policy information about availability of data

All manuscripts must include a data availability statement. This statement should provide the following information, where applicable:

- Accession codes, unique identifiers, or web links for publicly available datasets
- A description of any restrictions on data availability
- For clinical datasets or third party data, please ensure that the statement adheres to our policy

Source data are provided with this paper. All data are available in the main text, supplementary information, and/or source data. All materials used in the investigation are available upon request to corresponding authors. Databases used to retrieve mf-Lon gene and protein sequences were NCBI (accession number KM521209) and UniProt (accession number Q6F160), respectively. Saccharomyces genome database was used to obtain sequence information for PIF1, APN1, and MIP1 genes.

Research involving human participants, their data, or biological material

Policy information about studies with human participants or human data. See also policy information about sex, gender (identity/presentation), and sexual orientation and race, ethnicity and racism.

Reporting on sex and gender	N/A
Reporting on race, ethnicity, or other socially relevant groupings	N/A
Population characteristics	N/A
Recruitment	N/A
Ethics oversight	N/A

Note that full information on the approval of the study protocol must also be provided in the manuscript.

Field-specific reporting

Please select the one below that is the best fit for your research. If you are not sure, read the appropriate sections before making your selection.

☒ Life sciences ☐ Behavioural & social sciences ☐ Ecological, evolutionary & environmental sciences

For a reference copy of the document with all sections, see nature.com/documents/nr-reporting-summary-flat.pdf

Life sciences study design

All studies must disclose on these points even when the disclosure is negative.

Sample size

For estimation of mitochondrial GFP-PDT degradation in yeast (Figure 1b-c) - at least 600 cells from each sample were analysed.

For estimation of cytosolic GFP-PDT degradation in yeast (Figure 1d-e) - at least 100 cells from each sample were analyzed.

For estimation of degradation of mitochondrial BFP-PDT in human cells (Figure 5) - at least 40 cells from each condition were analyzed.

For evaluation of mitochondrial nucleoid organization (Supplementary Figure 3b-f) - at least 630 cells from each sample were analysed.

For estimation of mito-GFP-PDT degradation in pim1::mf-Lon expressing cells (Supplementary Figure 4d) - at least 150 cells were analyzed.

Data exclusions

For quantification of yeast mtDNA signals (Supplementary Figure 3), cells that had area less than 10 µm2 or circularity less than 0.85 were excluded from analysis. To exclude noise/debris and occasional weakly stained nucleus DNA, spots that displayed area less than 0.06 µm2 or median integrated density less than 500 a.u. (arbitrary units) were excluded from analysis. For quantification of U2OS cells, cells where the

area masked in mfLON or BFP/BFP-PDT channels, was below 10µm2, were excluded from analysis. In addition, the outliers were indentified by IQR procedure and subsequently excluded from statistical analysis.

Replication

Each experiment was initially performed with at least two independent yeast colonies. After this, a single colony was randomly chosen and the experiment was repeated at least three independent times, with the following exceptions
1. Fig 4b (WB for Pif1-PDT), repeated 2 times.
2. Extended Fig 1c (WB for GFP-PDT), repeated 2 times.
3. Extended Fig 3i (WB for Hsp60), repeated 2 times
Extended Fig 4d-e (Visualization of GFP-PDT degradation in pim1del::mito-mf-Lon cells), repeated 2 times.

The transfections experiments with the U2OS cells were repeated 3 times.

All reproductions were successful and consistently yielded presented data.

Randomization

The strains/cell lines employed in the research were isogenic, therefore genetic manipulation inherently introduces a level of randomness.

After genetic manipulation, the strain types were known. Positive and negative controls were always used to ensure that genetic manipulation worked and were assigned as "experimental control group" (eg cells expressing WT GFP, or cells that received no transforming/transfecting DNA). The "test group" (eg cells expressing Lon and GFP- or BFP-PDT) were examined for the phenotype (here, microscopic analysis of GFP/BFP signals).

When fluorescent signals were assessed, we randomly selected yeast cells in the brightfield channel and moved to the GFP channel to asses the degradation. Similarly, areas containing transfected human U2OS cells were randomly selected in in tdTomato channel (when BFP or BFP-PDT was transfected) or GFP channel (when only mfLON was transfected), and then assessed for BFP-PDT degradation concomitantly with mf-Lon expression.

Blinding

Blinding is not applicable in such study design. This is because for experiments with genetic manipulation of isogenic strains/cell lines, which can be confirmed by phenotypic and sequence analysis, it is not possible to introduce bias.

Reporting for specific materials, systems and methods

We require information from authors about some types of materials, experimental systems and methods used in many studies. Here, indicate whether each material, system or method listed is relevant to your study. If you are not sure if a list item applies to your research, read the appropriate section before selecting a response.

Materials & experimental systems

n/a	Involved in the study
☐	☒ Antibodies
☐	☒ Eukaryotic cell lines
☒	☐ Palaeontology and archaeology
☒	☐ Animals and other organisms
☒	☐ Clinical data
☒	☐ Dual use research of concern
☒	☐ Plants

Methods

n/a	Involved in the study
☒	☐ ChIP-seq
☒	☐ Flow cytometry
☒	☐ MRI-based neuroimaging

Antibodies

Antibodies used

anti-HA antibody (Sigma, cat# 11666606001, dilution 1:1000),
mf-Lon - anti-Flag (Sigma-Aldrich, cat# F1804-5 MG, dilution 1:3000),
histone - anti-H3 (Abcam, cat# ab1791, dilution 1:1000),
Cox2 – anti-MTCO2 (Abcam, cat# ab110271, dilution 1:1000)
GFP - anti-GFP (Abcam, cat# ab6556, dilution 1:1000)
HSP60 – anti-HSP60 monoclonal antibody LK2 (AH Diagnostics, cat# ADI-SPA-807-E, dilution 1:1000),

The following antibodies were gifts from Martin Ott:
Mrpl4 – anti-Mrpl4 (dilution 1:2000),
Mrpl36 – anti-Mrpl36 (dilution 1:500),
Mrpl40 – anti-Mrpl40 (dilution 1:2000)
Mdh1 – anti-Mdh1 (dilution 1:10000)

Secodary antibodies used were:
Polyclonal Goat Anti-Rabbit Immunoglonulins/HRP (DAKO, cat# P0448, dilution 1:5000)
used for antibodies against Mrpl4 , Mrpl36, Mrpl40, Mdh1, H3, GFP

Goat Anti-Mouse Immunoglobulins/HRP (DAKO, cat# P0447, dilution 1:5000, with exception in bracket below)
used for antibodies against HA, FLAG (1:10000), Cox2, Hsp60.

Validation

Commercially used antibodies were validated as described in the manufaturer's website. The certificate of analysis, product sheet and publications citing individual antibodies can be found in the following links:
anti-HA antibody (https://www.sigmaaldrich.com/SE/en/product/roche/roaha)
mf-Lon - anti-Flag (https://www.sigmaaldrich.com/SE/en/product/sigma/f1804),
histone - anti-H3 (https://www.abcam.com/en-at/products/primary-antibodies/histone-h3-antibody-nuclear-marker-and-chip-grade-ab1791),
Cox2 – anti-MTCO2 (https://www.abcam.com/products/primary-antibodies/mtco2-antibody-epr3314-ab79393.html)
GFP - anti-GFP (https://www.abcam.com/en-be/products/primary-antibodies/gfp-antibody-ab6556)
HSP60 – anti-HSP60 monoclonal antibody LK2 (https://www.enzolifesciences.com/ADI-SPA-807/hsp60-monoclonal-antibody-lk-2/).

Antibodies obtained from Martin Ott lab were validated in-house, and have been published in:
"Prestele, M., Vogel, F., Reichert, A. S., Herrmann, J. M. & Ott, M. Mrpl36 Is Important for Generation of Assembly Competent Proteins during Mitochondrial Translation. Mol Biol Cell 20, 2615–2625 (2009)",
"Gruschke, S. et al. Proteins at the Polypeptide Tunnel Exit of the Yeast Mitochondrial Ribosome*. J Biol Chem 285, 19022–19028 (2010)", and
"Toth, A. et al. Membrane-tethering of cytochrome c accelerates regulated cell death in yeast. Cell Death Dis 11, 722 (2020)".

Eukaryotic cell lines

Policy information about cell lines and Sex and Gender in Research

Cell line source(s)

Yeast strains of W303 background which was present in the Björkegren lab yeast stock.

U-2 OS primary cell lines used in the study are commercially available (ATCC HTB-96) and were derived from female patient with osteosarcoma (https://www.atcc.org/products/htb-96).

Authentication

The human cell lines were not authenticated by us.

The yeast W303 strains, both wild type and transformed, were always checked for expected markers to ensure that they were correct.

Mycoplasma contamination

The cell lines were not tested for mycoplasma contamination .

Commonly misidentified lines
(See ICLAC register)

No commonly misidentified lines were used in the study.

Plants

Seed stocks

N/A

Novel plant genotypes

N/A

Authentication

N/A
